# Evaluation of saliva self-collection devices for SARS-CoV-2 diagnostics

**DOI:** 10.1186/s12879-022-07285-7

**Published:** 2022-03-25

**Authors:** Orchid M. Allicock, Mary E. Petrone, Devyn Yolda-Carr, Mallery Breban, Hannah Walsh, Anne E. Watkins, Jessica E. Rothman, Shelli F. Farhadian, Nathan D. Grubaugh, Anne L. Wyllie

**Affiliations:** 1 Department of Epidemiology of Microbial Diseases, Yale School of Public Health, New Haven, CT 06510 USA; 2 Department of Medicine, Section of Infectious Diseases, Yale School of Medicine, New Haven, CT 06510 USA

## Abstract

**Background:**

There is an urgent need to expand testing for SARS-CoV-2 and other respiratory pathogens as the global community struggles to control the COVID-19 pandemic. Current diagnostic methods can be affected by supply chain bottlenecks and require the assistance of medical professionals, impeding the implementation of large-scale testing. Self-collection of saliva may solve these problems, as it can be completed without specialized training and uses generic materials.

**Methods:**

We observed 30 individuals who self-collected saliva using four different collection devices and analyzed their feedback. Two of these devices, a funnel and bulb pipette, were used to evaluate at-home saliva collection by 60 individuals. SARS-CoV-2-spiked saliva samples were subjected to temperature cycles designed to simulate the conditions the samples might be exposed to during the summer and winter seasons and sensitivity of detection was evaluated.

**Results:**

All devices enabled the safe, unsupervised self-collection of saliva. The quantity and quality of the samples received were acceptable for SARS-CoV-2 diagnostic testing, as determined by human RNase P detection. There was no significant difference in SARS-CoV-2 nucleocapsid gene (N1) detection between the freshly spiked samples and those incubated with the summer and winter profiles.

**Conclusion:**

We demonstrate inexpensive, generic, buffer free collection devices suitable for unsupervised and home saliva self-collection.

**Supplementary Information:**

The online version contains supplementary material available at 10.1186/s12879-022-07285-7.

## Introduction

Over a year since COVID-19 was declared a pandemic, the demand for testing remains high. Even with the rollout of several vaccines, successful control strategies still depend upon the availability of reliable, scalable testing programs. Self-collection of saliva for SARS-CoV-2 testing can facilitate these demands. Numerous studies have shown that saliva is an equally sensitive substrate for the detection of SARS-CoV-2 RNA as nasopharyngeal swabs [1–5]. Unlike sampling with nasopharyngeal swabs, self-collection of saliva is non-invasive and does not require specialized training to perform [6]. Moreover, SARS-CoV-2 RNA is stable in saliva at a broad range of temperatures and for an extended period of time, obviating the need for cold chain storage and preservatives or buffers that increase the costs of collection [7].

While saliva has been used as a diagnostic testing substrate for pathogenic antibodies [8–10], its utility in viral pathogen detection has been limited to viruses like human immunodeficiency virus [11], measles, mumps, and rubella [12], human papillomavirus [13], Epstein–Barr virus [14] and certain viral co-infections [15–17], all strictly in research settings. Before 2020, the only PCR-based diagnostic test using saliva (saliva swabs) approved or authorized by the U.S. Food and Drug Administration (FDA) was for the detection of human cytomegalovirus in babies [18]. Through the development of saliva-based diagnostic tests, COVID-19 testing became more accessible.

Despite its advantages, if saliva is collected improperly, it is difficult to handle in the laboratory [19]. Improper self-collection may also pose a safety risk if potentially biohazardous materials are mishandled. Therefore, it is essential that self-collection of saliva is safe and can produce testable samples. Equally important is establishing the acceptability of self-collection among the public because methods that are deemed uncomfortable, difficult, or confusing are unlikely to gain traction in the population.

**Fig. 1:**
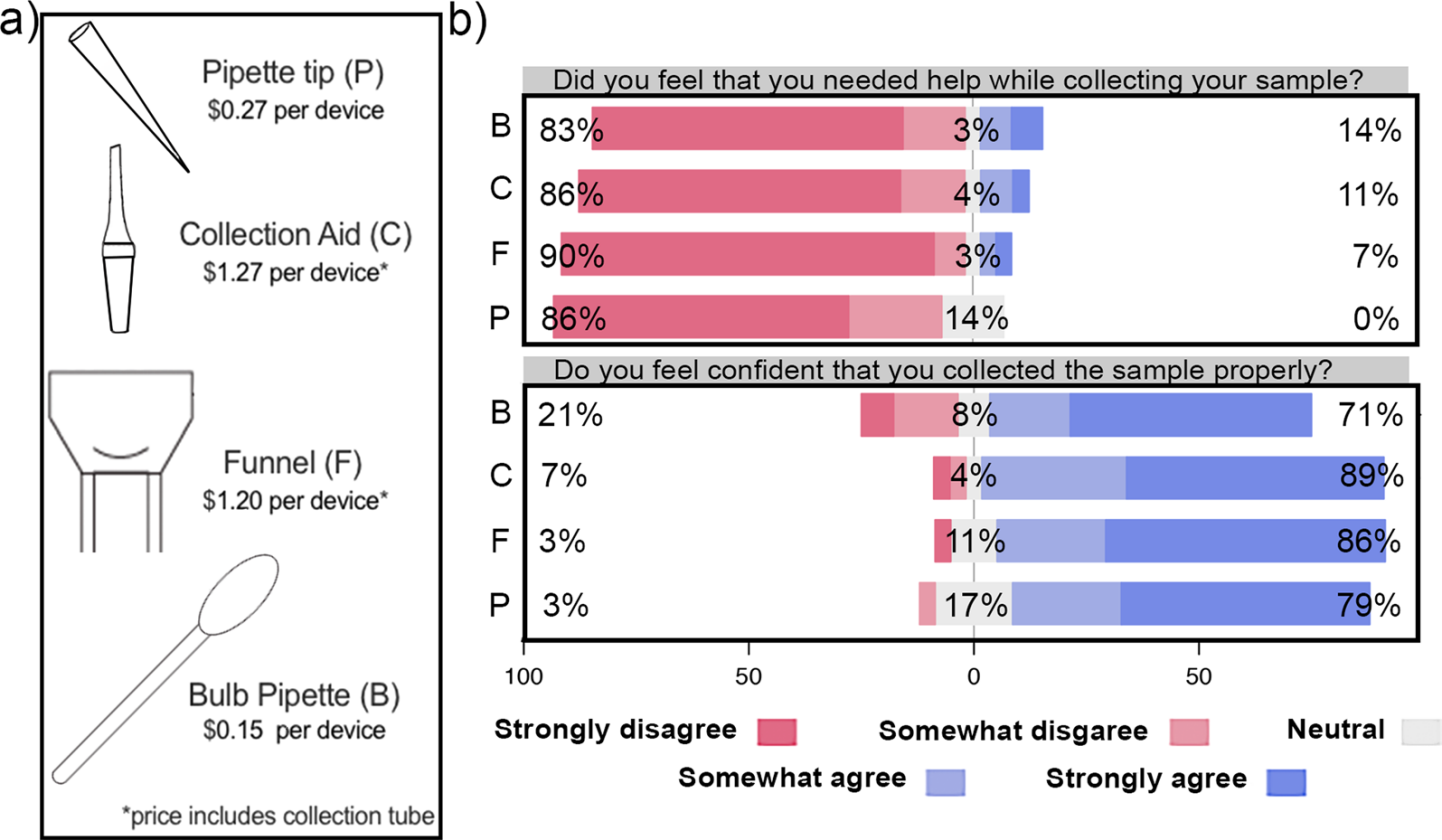
Collection devices are inexpensive, easy to use, and yield testable samples. Survey responses were reported from strongly disagree to strongly agree. **a** The four collection devices tested are inexpensive and provide users with a range of features to choose from. Prices at time of publication are shown in US dollars. **b** Participants reported being self-sufficient and confident in their ability to correctly collect saliva samples (from Additional file 2: Fig. S2). The questions are displayed above the corresponding graphs. The percentage response value for each device is shown above each bar. Two sets of participant responses were excluded because one participant did not provide a response for all four devices and one did not understand the response scale. *P* pipette tip, *C* collection aid, *F* funnel, *B* bulb pipette

In this study, we evaluated the experience of thirty individuals who self-collected saliva using four different saliva collection devices: a P1000 pipette tip, a Salimetrics Saliva Collection Aid (Salimetrics LLC, Pennsylvania, USA), a funnel, and a bulb pipette (Fig. 1a). We found that all four devices enabled the consistent and safe collection of true saliva that was acceptable for SARS-CoV-2 diagnostic testing with the SalivaDirect RT-qPCR-based assay. Using this information, we then evaluated the suitability of both a funnel and a bulb pipette for unsupervised at-home saliva collection. Our findings demonstrate the suitability of multiple device options for use in saliva self-collection kits. This variety not only helps to avoid supply chain bottlenecks but could also promote broader acceptance of this method by improving the ease of self-collection and of sample processing in the laboratory.

## Methods

### Ethics

This study was conducted in accordance with an Institutional Review Board protocol reviewed and approved by the Yale University Human Research Protection Program (IRB Protocol ID: 2000028394).

### Study design

For the initial evaluation of unobserved saliva collection, 30 participants between the ages of 20 and 80 years were enrolled. Individuals who had previously provided a saliva sample, who had relevant, career-level laboratory experience, or who were experiencing symptoms of respiratory infection were excluded from enrollment. Once informed consent was provided, participants received a collection kit containing the four saliva collection devices (Fig. 1a), corresponding collection instructions, a biohazard bag, and five alcohol wipes. Participants self-collected four saliva samples consecutively and in a randomized order. Members of the study team observed these collections via a video platform with minimal interaction with the study participant. The observer turned off video and audio on their device for the duration of the four collections and provided no instructions on sample collection. Following each collection, both the observer and the study participant completed a survey about the experience, scoring responses on a scale of 1 (strongly disagree) to 5 (strongly agree) (Additional file 1).

**Fig. 2:**
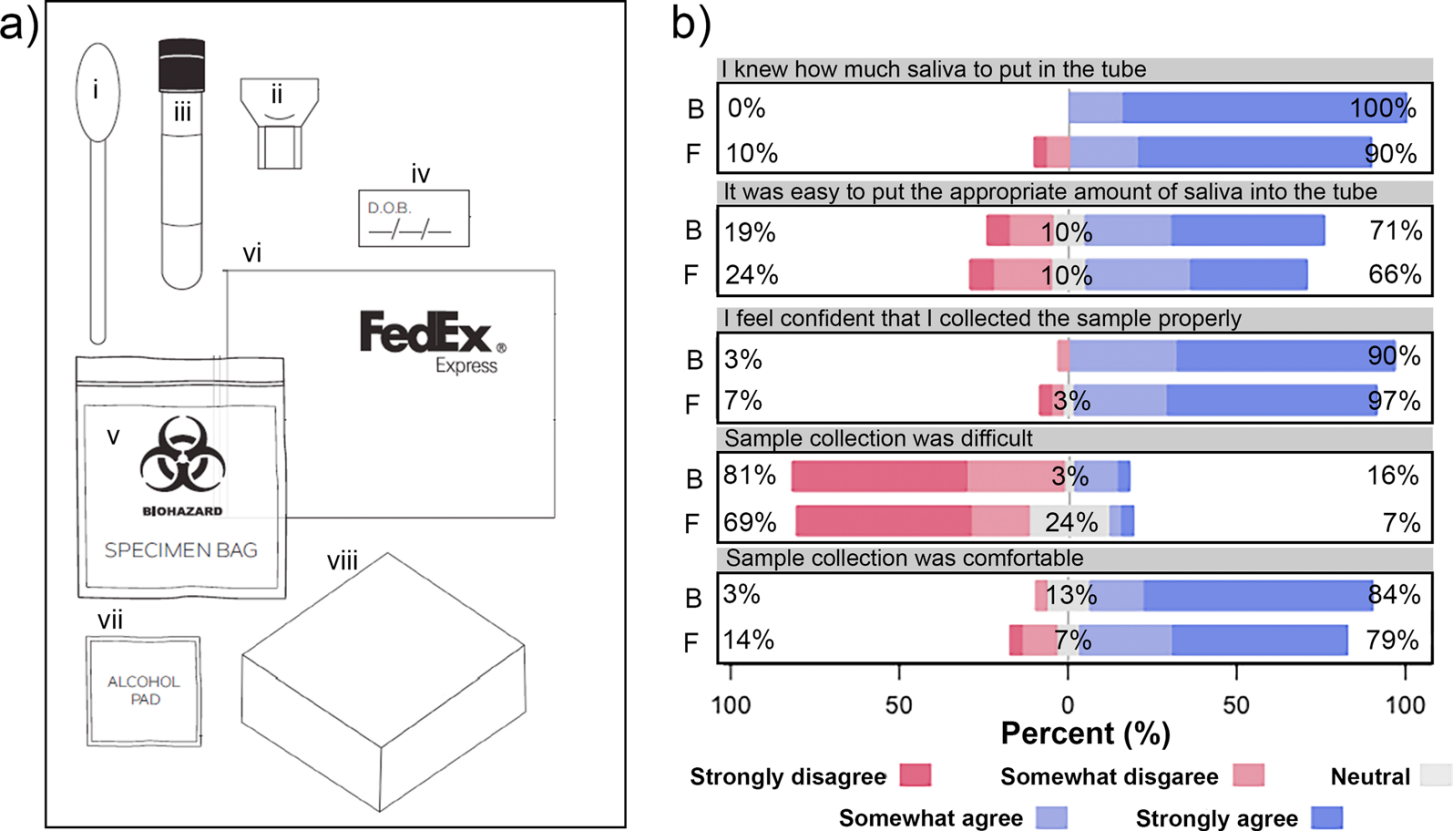
At-home saliva collection kit components suitable for sample collection. **a** Each of the participants were sent an at-home collection kit comprised of either a funnel (i) or bulb pipette (ii) with a labeled screw-cap tube (iii), patient identifier sticker (iv), biohazard collection bag with absorbent sheet (v), FedEx UN 3373 Pak (vi), an alcohol pad (vii), and box for return shipment (viii). **b** Participant confidence in at-home self-collection of saliva when using either a funnel or bulb pipette (from Additional file 4: Fig. S4). Survey responses were reported on a scale of 1 (strongly disagree) to 5 (strongly agree). Overall, there was no significant difference between the collection devices in relation to the participant’s confidence and ability to use either device. The questions are displayed above the corresponding graphs. *F* funnel, *B* bulb pipette

An additional 60 participants were recruited into the study through an online, social media post to evaluate unsupervised at-home saliva collection. Participants were required to be at least 18 years of age, reside in the contiguous United States with no previous experience with providing saliva for diagnostic testing. Participants provided demographic data and were consented via an online form to limit direct contact with study participants, and to replicate an unsupervised at-home collection as closely as possible. Study participants were selected from consenting individuals to ensure a diverse range of age and race. Study participants were mailed an at-home self-collection kit containing a saliva collection device, a collection tube, collection instructions (Additional file 9), a biohazard bag, an alcohol wipe, and a FedEx envelope for sample return (Fig. 2a). Samples returned to the laboratory were stored at 4 °C for up to 4 days until testing.

### Sample testing

All saliva samples (n = 183) were tested for a region of the SARS-CoV-2 nucleocapsid gene (N1) and human RNase P gene (RP) using the SalivaDirect protocol [20]. A laboratory survey assessing the quality of each sample was completed by the technician during testing.

### Simulated shipping conditions

To evaluate the stability of SARS-CoV-2 RNA detection following the shipment of saliva samples, saliva from healthy individuals were pooled and spiked with a clinical saliva sample containing a known concentration of SARS-CoV-2 viral particles (3.7 × 10^4^ copies/µL) [3] and diluted to 50 and 12 virus RNA copies/μL. As recommended by the FDA, the samples were cycled through temperatures typically expected when samples are shipped during the summer and winter [21]. The summer profile consisted of incubating the spiked saliva samples at 40 °C for 8 h, RT for 4 h, 40 °C for 2 h, 30 °C for 36 h, then 40 °C for 6 h. The winter profile consisted of exposing the sample to − 20 °C for 8 h, RT for 4 h, − 20 °C for 2 h, 4 °C for 36 h, then − 20 °C for 6 h. Spiked saliva samples (50 and 12 virus RNA copies/μL) were also incubated at 50 °C for 24 h. After incubation, the samples were tested for the presence of SARS-CoV-2 RNA by the SalivaDirect protocol.

### Statistical analysis

Participant, observer, and laboratory survey questions were tested for internal reliability with Cronbach’s alpha using R v.4.0.2. Significant statistical differences across the four devices were calculated using one-way ANOVA. Participants who did not provide a response for all four devices were excluded from the analysis for the corresponding question (maximum of 6 for question 10). For the laboratory surveys, responses to questions 2, 3, and 4 were identical across devices and therefore could not be assessed using one-way ANOVA. For the at-home self-collection of saliva, the differences between the bulb pipette and funnel kits were assessed using the Mann–Whitney test. The differences in SARS-CoV-2 RNA stability kept at profiles were assessed using the Kruskal–Wallis test, and multiple comparisons were corrected with Dunn’s test. All of the analysis described above was done in GraphPad v.9.1.0.

## Results

*All four saliva collections devices were deemed usable by the study participants, but individual preference influenced their relative acceptability.* We aimed to enroll participants who represented a range of racial and educational backgrounds (Table 1). In 100% of the observed collections, study participants appeared confident in their ability to complete the collection correctly (Fig. 1b). The majority of participants (93%) understood the importance of following the instructions carefully to avoid incorrect test results, and during only two collections (1.67%), participants appeared to not adequately follow these instructions for proper sample collection (Additional file 2: Fig. S2b).

**Table 1:** Demographic characteristics of the participants from the pilot study and the at-home collection kit study

Category	Pilot study (n = 30)	At-home kit (n = 60)		
	n (%)	Overall (%)	Bulb (%, n = 31)	Funnel (%, n = 29)
Sex				
Male	11 (37)	28 (47)	14 (45)	14 (48)
Female	19 (63)	32 (53)	17 (55)	15 (52)
Age				
18–29	7 (23)	13 (22)	7 (23)	6 (21)
30–39	16 (53)	27 (45)	11 (35)	16 (16)
40–49	4 (13)	5 (8)	4 (13)	1 (3)
50–59	0 (0)	5 (8)	3 (10)	2 (7)
60–69	1 (3)	6 (10)	4 (13)	2 (7)
70+	2 (7)	4 (7)	2 (6)	2 (7)
Education				
High School/GED	2 (7)	15 (25)	9 (29)	6 (21)
Bachelors	7 (23)	21 (35)	11 (35)	10 (34)
Masters	10 (33)	11 (18)	7 (23)	4 (14)
Ph.D/MD	11 (37)	13 (22)	4 (13)	9 (31)
Race				
Black/African American	4 (13)	10 (17)	6 (19)	4 (14)
Hispanic/Latino	4 (13)	10 (17)	5 (16)	5 (17)
Asian/South Asian	6 (20)	7 (12)	4 (13)	3 (10)
White	15 (50)	33 (55)	16 (52)	17 (59)
Native American	1 (3)	0 (0)	0	0

Of the 10 participant survey questions, only Question 5 (“Was collecting the sample difficult in general?”) varied statistically significantly across devices; however, this question was found to not be internally reliable (Additional files 6 and 7). In this case, the bulb pipette scored the least favorably (mean = 3.1) compared to the other devices (pipette tip, mean = 2; funnel, mean = 2.3; collection aid, mean = 1.7) (Additional file 2). Participants commented that the bulb pipette introduced bubbles and caused discomfort if it suctioned the inside of their mouth (Table 2).

**Table 2:** Participant observations and experiences with the unobserved collection

Comments from pilot study			
Funnel	Bulb pipette	Pipette tip	collection aid
“Very easy!”	“Least favorite.”	“Easiest so far.”	“Liked the most.”
“Huge tube but clean.”	“Lots of work for what felt like little yield.”	"Messy.”	“Was a little gross”
“I think this is the best one.”	“Fav[orite] and way quickest method.”	“Bubbled up not sure what to do.”	“Most difficult.”
“Slow.”	“It solved the spilling issue but created too much bubbles which makes it difficult to fillfull [sic] the amount.”	“The saliva was getting stuck at the pipette tip making it difficult to pass through.”	“Easiest.”
“2nd easiest.”	“Kept suctioning in mouth instead of saliva, created bubbles and did not collect saliva [efficiently].”	“Least fav[orite] collection [because] saliva go [sic] everywhere”	“Favorite collection method but risk of saliva dribble.”

Comments from at-home collection kits	
Funnel	Bulb pipette
“No clear step to unscrew the collection tube and attach the funnel.”	“I wanted pictures for each step to make sure I was doing it right.”
“The directions never said to take off the cap to the tube.”	“The directions were not clear at all.”
“It took me a little bit to understand that the sample goes back in the box and then goes in the [FEDEX] bag. Also, it never specifically said to unscrew the tube and attach the funnel.”	“It should have been made clear that the specimen goes in the box and then the package.”
“[I] had to collect a lot of saliva (half of a large tube)—that took a while. The font in the instructions could be larger.”	“The font was too tiny, impossible to read…Transferring the saliva from the pipette to the little tube was challenging (the tube opening was too small).”

Survey comments from participants from pilot study and the at-home collection kits. The comments presented here are representative but not exhaustive

*No.* number

Despite this feedback, all participants provided a sufficient volume of saliva for testing with all four devices, the majority did not think they required assistance during the sample collection (93%), and in only 18 collections (16%), participants did not feel confident that they had collected the sample correctly with the bulb pipette (Fig. 1b). Similarly, observers reported that the majority of participants did not appear to struggle with the collection process (115/120, 95.8%, Additional file 2: Fig. S2b).

In addition to answering the survey questions, participants were given the opportunity to provide general feedback. Each device received a range of comments from participants reflecting differences in personal preference (Table 2). For example, though the bulb pipette received the largest number of negative comments (n = 11), one participant stated it was their favorite of the four devices. Interestingly, there was no general consensus around an overall preferred device; however, the size of the devices was a common theme among participant feedback. Some participants (4/30, 13%) found the pipette tip and collection aid to be too small, whereas the large size of the funnel and its collection tube were noted to be an advantage. More research is needed to determine which types of devices may be most suitable for specific demographic groups, but it is likely that providing a range of options will promote the general acceptability of saliva self-collection for pathogen diagnostic testing.

*Unsupervised saliva collection can be reliably conducted at home*. In order to achieve diversity in the demographics of the participants, we selected 84 of the 246 participants who consented to unsupervised at-home saliva collection study, based on age, sex, race and educational status. The participants were sent self-collection kits containing either a funnel (n = 43) or bulb transfer pipette (n = 41) to aid saliva collection (Fig. 2a). Of those distributed, 66 kits were returned, however six participants did not complete the survey, so were excluded from the study. Overall, survey responses following unsupervised collection were favorable (Fig. 2b). Participants reported feeling confident with carrying out self-collection properly and that the process was not difficult.

Importantly, study participants clearly understood the required process of sample collection, with 100% of participants acknowledging that they understood not to eat/drink/smoke prior to collecting the sample, and 88.33% understood that incorrect sampling could result in false results (Additional file 4). There were slight differences in the user experience between bulb pipette kits and the funnel kits; 16% of the participants found that the sample collection was difficult with the bulb pipette as compared to only 7% of the participants using the funnel.

*Self-collection of saliva was safe and yielded testable samples*. Ensuring the proper handling of potentially biohazardous material is an essential consideration for saliva self-collection to be implemented on a large scale. Specifically, contamination of the collection tube with virus-infected saliva poses the greatest health and safety risk for this method.

Some participants did contaminate the outside of their collection tubes with saliva during the pilot collection (27.8%) and the at-home kit study (21.7%), but participants from the pilot study were observed sanitizing the collection tube with an alcohol wipe in accordance with the provided instructions and the majority of at-home study participants reported understanding what to do in this situation. Additionally, as directed in the written instructions, 87% of participants in the pilot study washed or sanitized their hands before and after completing the collections. Regardless, strict sample handling safety precautions should be applied by all testing laboratories when receiving any clinical sample type.

**Fig. 3:**
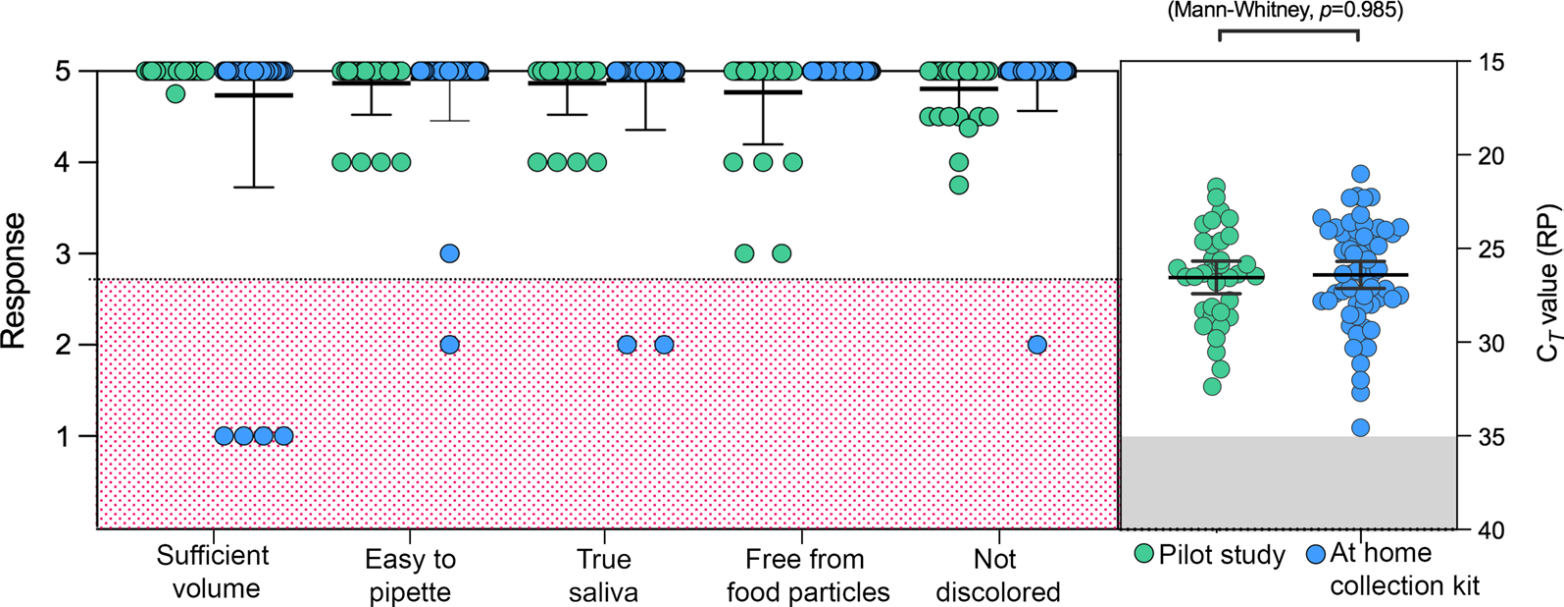
The quality of the samples was adequate for testing with a PCR-based assay. Laboratory survey questions pertaining to the quality of the samples are shown on the x-axis (from Additional files 3 and 5). Data points represent the mean response, green dots represent samples collected from the pilot study and blue dots represent samples collected from the at-home collection kit. Survey responses were reported on a scale of 1 (strongly disagree) to 5 (strongly agree). Samples with less favorable responses are highlighted in red. Mean and standard deviation (st. dev.) are shown in black. The graph on the right shows the cycle threshold (Ct) values for the internal control RNAse P (RP) from each of the saliva samples submitted. The dots represent Ct value per participant. Ct values over 35 are considered invalid and are highlighted in gray. P-value is shown using one-way Mann–Whitney. Mean and standard deviation (st. dev.) are shown in black

Our secondary objective was to compare the quality of samples collected using each device. We found that all of the samples received (both unobserved as well as unsupervised at-home self-collection) were of sufficient quality for testing with SalivaDirect, demonstrating how true saliva, which naturally pools in the mouth, can be easily handled in the laboratory. Specifically, laboratory survey responses confirmed that 100% of the samples collected during the pilot study were easy to pipette and of sufficient volume (> 0.5 mL) (Fig. 3). Slight discoloration was noted in 18 samples (15%) and food particles were observed in 20 samples (5 participants, 16.7%), but these did not affect test results. No sample tested positive for SARS-CoV-2. The average cycle threshold (Ct) value for the negative control, RNAse P gene (RP), was within the typical range (23–28 Cts) [3] for the majority of samples from the pilot study (73%), indicating that the use of different collection methods did not interfere with the diagnostic assay (Fig. 3). We did not find a significant difference between matched samples across devices using one-way ANOVA (Additional file 3).

The overall quality of the saliva samples from the at-home kit study was also acceptable, but with slight differences between the two collection devices. Of the 60 samples returned, 6.7% (n = 4) contained less than 0.5 mL saliva (half of the 1 mL tube provided), all from participants given the bulb pipette collection kit (Fig. 3). Despite this, all four samples were sufficient for testing, containing 60–500 µL of saliva. Besides low volume, the quality of the samples collected with the bulb pipette was high, with 100% of samples easy to pipet, free from food particles, not discolored and consisting of only true saliva. On the other hand, while 100% of the samples returned from the participants with the funnel collection kit were of sufficient volume, 2/29 samples might not have been “true saliva” and as a result were difficult to pipet. In addition, one of the samples was slightly discolored.

### SARS-CoV-2 detection in saliva remains stable following summer and winter shipping conditions

**Fig. 4:**
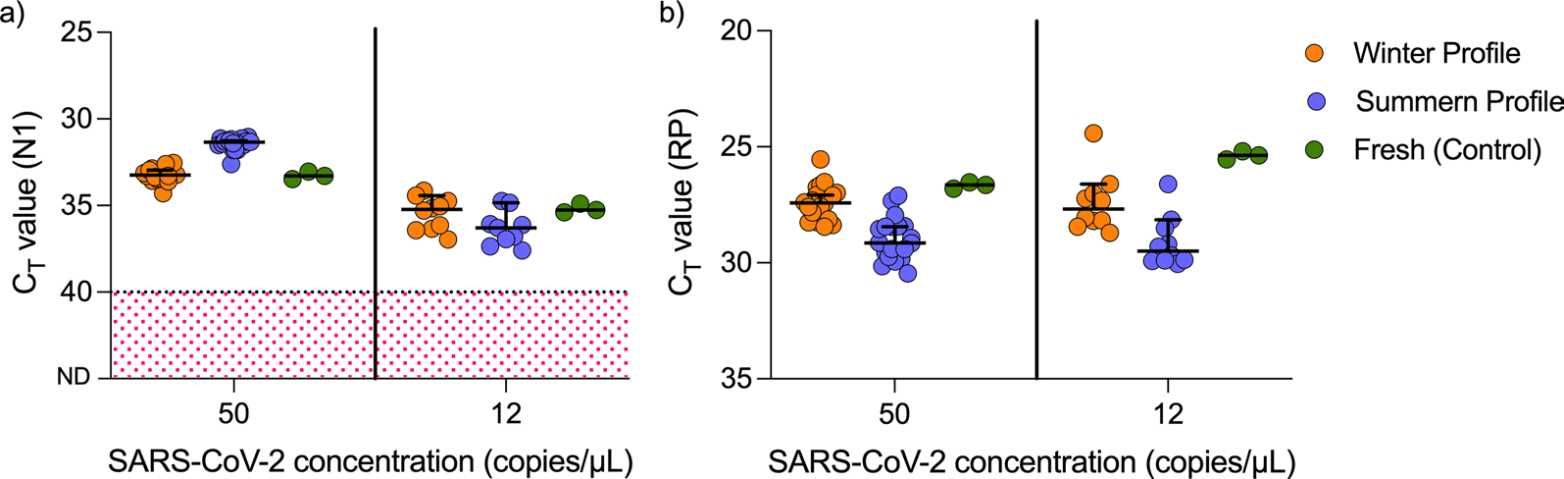
SARS-CoV-2 detection in saliva remains stable following summer and winter shipping conditions. Spiked saliva samples diluted twofold were incubated under summer or winter profiles in triplicate (from Additional file 8: Table S3). Summer profile consisted of 40 °C for 8 h, room temperature for 4 h, 40 °C for 2 h, 28 °C for 36 h, and 40 °C for 6 h. Winter profile consisted of − 20 °C for 8 h, room temperature for 4 h, − 20 °C for 2 h, 4 °C for 36 h, and − 20 °C for 6 h. Samples with invalid Ct values are highlighted in red. **a **No significant differences were found in N1 Ct values when comparing the profiles to the fresh (control) samples (Kruskal–Wallis; P > 0.05). **b** The Ct values for RP of samples for the winter profile were significantly higher than fresh samples (Kruskal–Wallis; P < 0.03). The horizontal bars indicate the median Ct value

We previously demonstrated that SARS-CoV-2 RNA detection in raw (unsupplemented) saliva remains relatively stable for prolonged periods over a range of temperatures (− 80 °C, 4 °C, ~ 19 °C or 30 °C) [7]. For the current study, we expanded upon this, and tested SARS-CoV-2-spiked saliva samples through temperature cycles designed to simulate the conditions the samples might be exposed to during the summer and winter seasons. Following the temperature cycles, SARS-CoV-2 nucleocapsid gene (N1) was reliably detected in saliva samples spiked with 50 SARS-CoV-2 copies/μL (20/20 replicates) and 12 SARS-CoV-2 copies/μL (9/10 replicates) (Fig. 4). There were no significant differences between N1 Ct values for the fresh samples and the samples incubated under summer (P > 0.99) or winter profiles (P > 0.99). However, the Ct values for human RNase P (RP) were significantly higher in the samples exposed to the winter profile (P < 0.03) when compared to fresh specimens, suggesting that RP RNA degraded over time (Additional file 5). This suggest that the N1 gene in saliva is stable when subjected to the temperatures expected during the transport of the samples in the summer and winter.

## Discussion

To combat the ongoing outbreaks of SARS-CoV-2, mass testing strategies which are cost-effective and free from supply chain disruptions are essential. Additional major barriers to frequent testing result from a need to schedule appointments at facilities staffed with trained personnel or testing aversion to swab-based methods. Scaling up the use of saliva self-collection as a routine diagnostic tool can expand access to testing for SARS-CoV-2 and could be reliably performed in workplaces, schools or college dormitories where regular testing is essential for safe day-to-day operations. To support these efforts, we aimed to identify saliva collection solutions with generic components without sacrificing the comfort of the participants or the effectiveness of collection. Results from this study demonstrate the usability and efficacy of several simple saliva collection methods for SARS-CoV-2 detection. Importantly, all of the devices promoted the collection of “true” saliva, which was acceptable for handling in the laboratory, and were deemed usable by our participants.

The data collected from the pilot study was used to inform our selection of the bulb pipette and funnel as the saliva collection devices for the at-home saliva collection kits. Though there was no clear preference in devices based on demographic factors like sex, education level, ethnicity or age, some of the older participants had issues with saliva collection using the bulb pipette. More studies can be done to specifically assess the usability of the collection devices in these specific populations. The availability of the option for unsupervised sample collection for COVID-19 testing could result in up to one-third more symptomatic persons seeking testing, especially in those populations of individuals who are at high risk for contracting the infection, or those who are unable/unwilling to go into clinical settings [22]. With more options available, individuals can select kits according to their needs and limitations.

We did not directly compare the self-collection process with the aid of a collection device to the process without a device, but the ability to collect true saliva in simple wide mouth tubes has been previously demonstrated [3, 23]. Wide-mouth tubes are not conducive for large-scale testing in labs with limited space or when sample processing requires the use of a liquid-handling robot, a piece of equipment present in most large clinical laboratories. Therefore, the collection devices we tested allow for an easy collection process into smaller tubes that are likely more amenable to the majority of laboratory procedures. Importantly, results from our study also demonstrate that these devices do not inhibit RNA-extraction free, RT-qPCR based diagnostic assays.

This study also evaluated the instructions for reliable saliva self-collection. The majority of the participants had no additional feedback, and the few comments we did receive were all related to the kit instructions, involving font size, mailing instructions and device assembly (see Table 2). This slight confusion was reflected in the participant survey responses, where 35% of participants were unsure of what to do if saliva came into contact with the outside of the tube and 26% were unsure of what to do if they had any questions. This feedback highlighted the need to further refine the instructions in order to decrease the likelihood of errors in saliva collection and improve the sample collection experience. Additionally, visual materials such as a video outlining the sample collection and shipping process could be helpful in future iterations of the kits.

This study was designed to evaluate the participant’s experience with self-collecting a saliva sample and returning it to a clinical lab for diagnostic testing, following our carefully developed instructions for use. While SARS-CoV-2 RNA was not detected in any of the samples collected, we have previously demonstrated the stability of SARS-CoV-2 RNA in simple laboratory plastic tubes (i.e., these plastics do not interfere with SARS-CoV-2 RNA detection). The potential for these devices has been demonstrated on a larger scale, with hundreds of thousands of K-12 students and faculty, regularly being tested with similar devices [24]. In the current study however, we demonstrate that these samples can be reliably collected outside of formal testing programs. In lieu of mailing clinical samples from confirmed COVID-positive individuals, we instead simulated expected shipping conditions in the laboratory and demonstrated that N1 detection at the limit of detection of the SalivaDirect assay remains stable.

While the sample size of the pilot study was small, and a majority of study participants held a college degree or higher, similar results were obtained when we enrolled a larger, more demographically diverse cohort for the unsupervised, at-home evaluation. It is important to note that we did not enroll individuals under the age of 18 and therefore cannot draw conclusions around the usability of these devices in children. However, large-scale pathogen surveillance testing involving self-collected saliva samples from school-aged children have been executed for SARS-CoV-2 and other pathogens (*Streptococcus pneumoniae)* [25, 26]. Also, the sample size was too small to determine if there are age-specific preferences in collection devices. More studies can be done to assess the utility of different collection devices in select populations.

## Conclusion

Even with ongoing vaccination campaigns, widespread, routine testing for SARS-CoV-2 will remain a staple of public health disease control strategies for at least another year. In this study we demonstrate inexpensive, generic, buffer free collection devices suitable for unsupervised and home saliva self-collection. The availability of unsupervised saliva collection as an option for COVID-19 diagnostics permits feasible, scalable, and affordable testing solutions. These findings led to the first FDA authorization for unobserved, at-home self-collection of raw, unsupplemented saliva for SARS-CoV-2 detection.

## Supplementary Information

**Additional file 1: Figure S1.** Survey questions posed to participants, observers, and laboratory personnel. For the pilot study, parts a), b) and c) were used. For the at-home kit, only a) and c) were used. Responses were given on a scale of 1 (strongly disagree) to 5 (strongly agree).

**Additional file 2: Figure S2.** Responses to participant and observer surveys (related to Fig. 1). Mean and standard deviation are marked in pink. Survey data were analyzed using one-way ANOVA. Responses to two questions (P5 and O5) differed significantly across devices and are denoted with black boxes. The numbers shown on the x-axis of those graphs are the mean response value.

**Additional file 3: Figure S3.** Responses to laboratory survey. P-values are shown for questions that could be assessed using one-way ANOVA. Mean and standard deviation (st. dev.) are shown for questions where responses were identical across devices.

**Additional file 4: Figure S4.** Responses to participant surveys for at home collection kit. Mean and standard deviation are marked in pink. Survey data were analyzed using Mann–Whitney. P < 0.05 is significantly different. F = funnel, B = bulb pipette.

**Additional file 5: Figure S5.** Responses to laboratory survey for at-home collection. Mean and standard deviation are marked in pink. P-values are shown for questions that could be assessed using Mann–Whitney. For L6, samples outside of the unacceptable Ct range (Ct > 35) is highlighted in gray. F = funnel, B = bulb pipette.

**Additional file 6: Table S1.** Internal reliability of survey question measured with Cronbach’s alpha.

**Additional file 7: Table S2.** Internal reliability for survey questions. The majority of internally reliable survey questions did not differ significantly across collection devices. (a) Analysis of responses for participant and observer survey questions found to be internally reliable with Cronbach’s alpha. (b) Analysis of laboratory survey responses.

**Additional file 8: Table S6.** Cycle threshold values for nucleocapsid and RNase P genes from simulated shipping data. Saliva samples were spiked at 50 and 12 SARS CoV-2 copies/µL, then processed fresh, with the summer or winter profiles.

**Additional file 9.** At home kit instructions. Instructions provided with the at-home collection kits.

## Data Availability

The datasets generated and/or analyzed during the current study are available in the Mendeley Data repository at https://data.mendeley.com/datasets/x2mv2ctm7c/1.

## Abbreviations

Ct: Cycle threshold
COVID-19: Corona virus disease, 2019
PCR: Polymerase chain reaction
SARS-CoV-2: Severe Acute Respiratory Syndrome Coronavirus 2
SD: Standard deviation
RNA: Ribonucleic acid
RP: RNase P gene
