# Supplementary material for: Evaluation of saliva self-collection devices for SARS-CoV-2 diagnostics
Source: BMC Infect Dis. 2022 Mar 25;22:284. doi: 10.1186/s12879-022-07285-7 (PMC8953967; doi:10.1186/s12879-022-07285-7)
Supplement: Supplementary file 1 — Additional file 1: Figure S1. Survey questions posed to participants, observers, and laboratory personnel. For the pilot study, parts a), b) and c) were used. For the at-home kit, only a) and c) were used. Responses were given on a scale of 1 (strongly disagree) to 5 (strongly agree). [file 12879_2022_7285_MOESM1_ESM.docx]

#### **Additional Information**

​​**(a)** Participant survey

**(b)** Observer survey


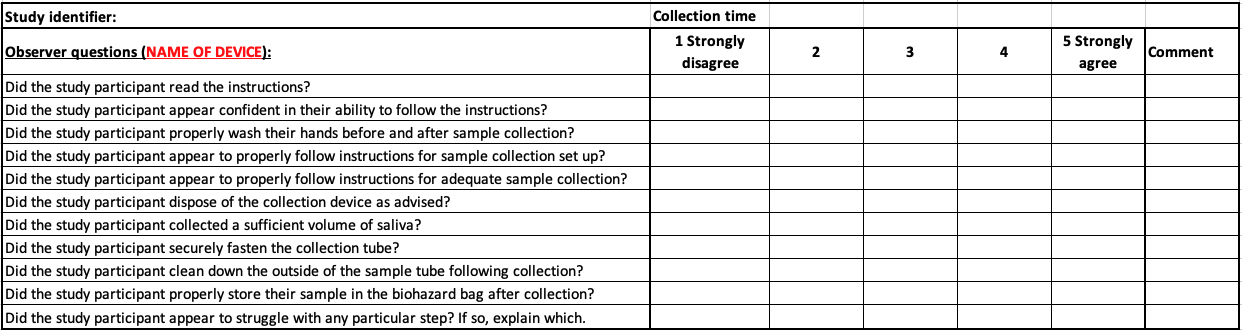


**(c)** Laboratory survey


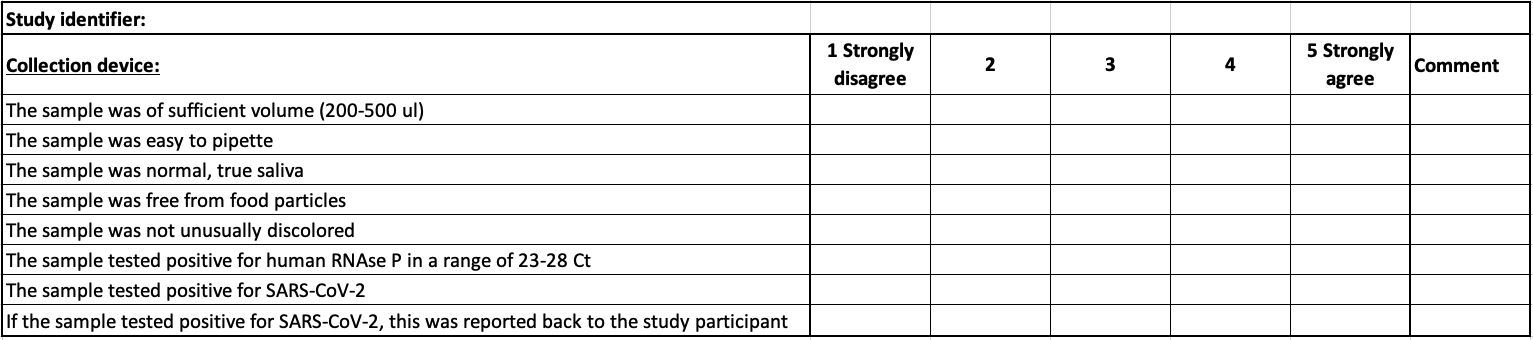


#### **Additional file 1: Figure S1 Survey questions posed to participants, observers, and laboratory personnel.** For the pilot study, parts a), b) and c) were used. For the at-home kit, only a) and c) were used. Responses were given on a scale of 1 (strongly disagree) to 5 (strongly agree).
