# Supplementary material for: Evaluation of saliva self-collection devices for SARS-CoV-2 diagnostics
Source: BMC Infect Dis. 2022 Mar 25;22:284. doi: 10.1186/s12879-022-07285-7 (PMC8953967; doi:10.1186/s12879-022-07285-7)
Supplement: Supplementary file 2 — Additional file 2: Figure S2. Responses to participant and observer surveys (related to Fig. 1). Mean and standard deviation are marked in pink. Survey data were analyzed using one-way ANOVA. Responses to two questions (P5 and O5) differed significantly across devices and are denoted with black boxes. The numbers shown on the x-axis of those graphs are the mean response value. [file 12879_2022_7285_MOESM2_ESM.docx]

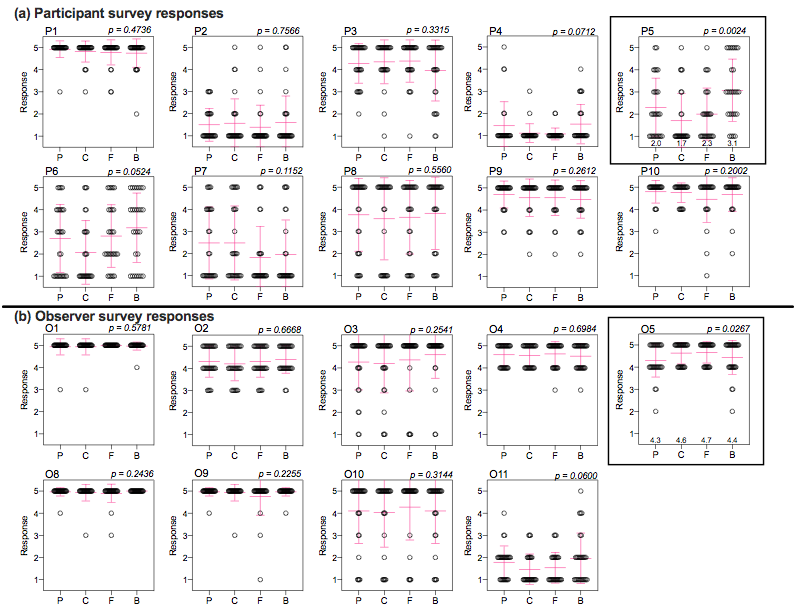


#### **Additional file 2: Figure S2 Responses to participant and observer surveys (related to Figure 1).** Mean and standard deviation are marked in pink. Survey data were analyzed using one-way ANOVA. Responses to two questions (P5 and O5) differed significantly across devices and are denoted with black boxes. The numbers shown on the x-axis of those graphs are the mean response value.
