# Supplementary material for: Evaluation of saliva self-collection devices for SARS-CoV-2 diagnostics
Source: BMC Infect Dis. 2022 Mar 25;22:284. doi: 10.1186/s12879-022-07285-7 (PMC8953967; doi:10.1186/s12879-022-07285-7)
Supplement: Supplementary file 3 — Additional file 3: Figure S3. Responses to laboratory survey. P-values are shown for questions that could be assessed using one-way ANOVA. Mean and standard deviation (st. dev.) are shown for questions where responses were identical across devices. [file 12879_2022_7285_MOESM3_ESM.docx]

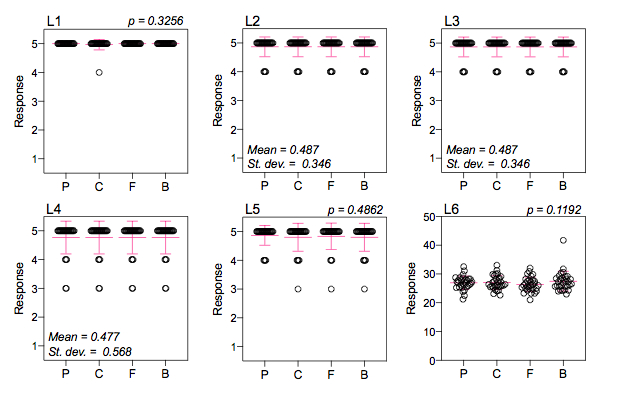


#### **Additional file 3: Figure S3 Responses to laboratory survey.** P-values are shown for questions that could be assessed using one-way ANOVA. Mean and standard deviation (st. dev.) are shown for questions where responses were identical across devices.
