# Supplementary material for: Evaluation of saliva self-collection devices for SARS-CoV-2 diagnostics
Source: BMC Infect Dis. 2022 Mar 25;22:284. doi: 10.1186/s12879-022-07285-7 (PMC8953967; doi:10.1186/s12879-022-07285-7)
Supplement: Supplementary file 4 — Additional file 4: Figure S4. Responses to participant surveys for at home collection kit. Mean and standard deviation are marked in pink. Survey data were analyzed using Mann–Whitney. P < 0.05 is significantly different. F = funnel, B = bulb pipette. [file 12879_2022_7285_MOESM4_ESM.docx]

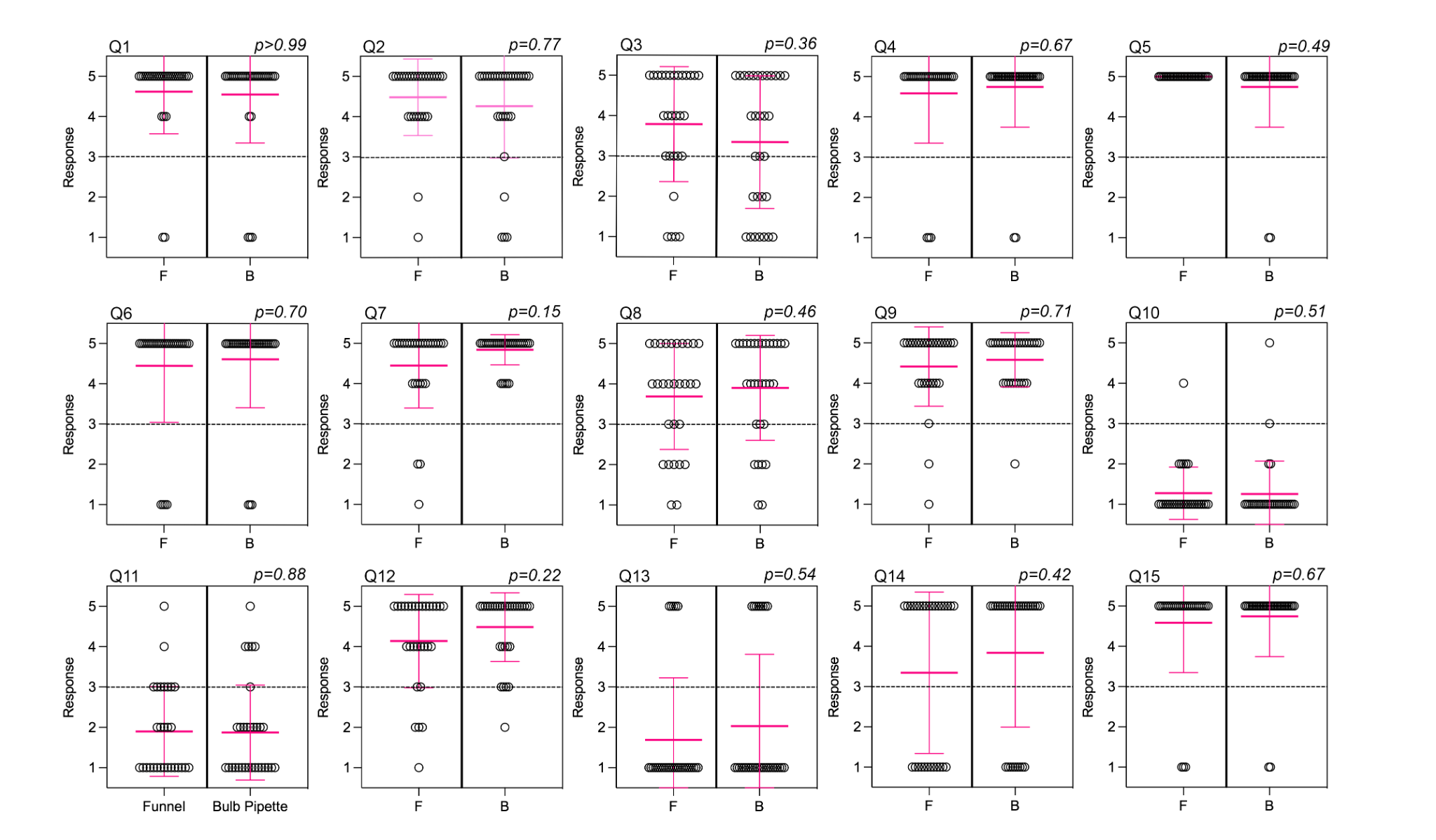


#### **Additional file 4: Figure S4 Responses to participant surveys for at home collection kit.** Mean and standard deviation are marked in pink. Survey data were analyzed using Mann-Whitney. P < 0.05 is significantly different. *Abbreviations: F = funnel, B = bulb pipette*
