# Supplementary material for: Evaluation of saliva self-collection devices for SARS-CoV-2 diagnostics
Source: BMC Infect Dis. 2022 Mar 25;22:284. doi: 10.1186/s12879-022-07285-7 (PMC8953967; doi:10.1186/s12879-022-07285-7)
Supplement: Supplementary file 5 — Additional file 5: Figure S5. Responses to laboratory survey for at-home collection. Mean and standard deviation are marked in pink. P-values are shown for questions that could be assessed using Mann–Whitney. For L6, samples outside of the unacceptable Ct range (Ct > 35) is highlighted in gray. F = funnel, B = bulb pipette. [file 12879_2022_7285_MOESM5_ESM.docx]

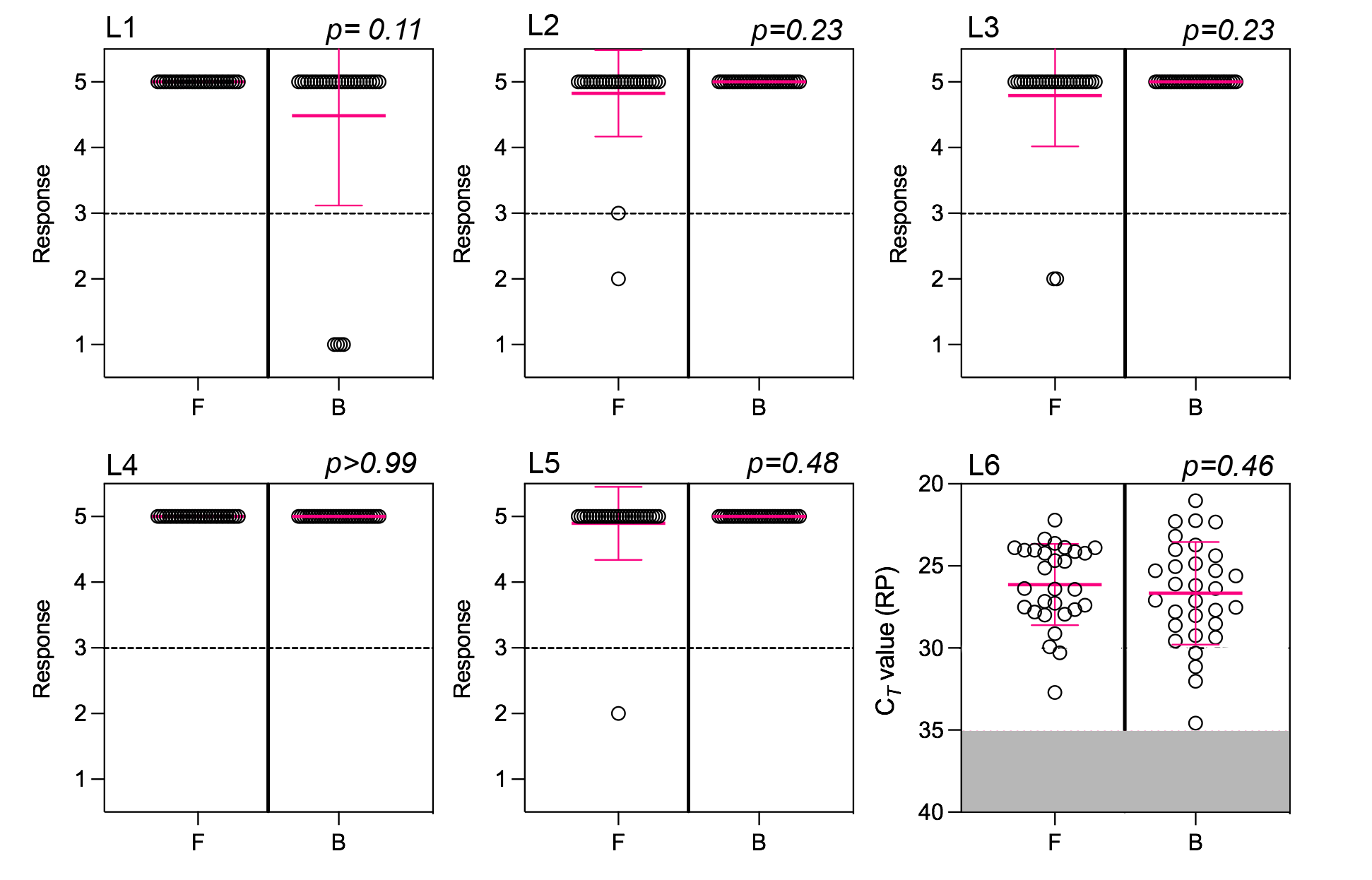


#### **Additional file 5: Figure S5 Responses to laboratory survey for at-home collection kit.** Mean and standard deviation are marked in pink. P-values are shown for questions that could be assessed using Mann-Whitney. For L6, samples outside of the unacceptable Ct range (Ct > 35) is highlighted in gray.
