# Supplementary material for: Evaluation of saliva self-collection devices for SARS-CoV-2 diagnostics
Source: BMC Infect Dis. 2022 Mar 25;22:284. doi: 10.1186/s12879-022-07285-7 (PMC8953967; doi:10.1186/s12879-022-07285-7)
Supplement: Supplementary file 9 — Additional file 9. At home kit instructions. Instructions provided with the at-home collection kits. [file 12879_2022_7285_MOESM9_ESM.pdf]

The Tempus + SalivaDirect **funnel collection kit**.

WARNING AND PRECAUTIONS BEFORE BEGINNING

Before providing your sample, first carefully read through the instructions below about how to provide a good saliva sample. **Not following the instructions could lead to an incorrect result.**

It is very important that you do not force yourself to produce a saliva sample. Please do not cough anything up from your chest. Please do not sniff anything back from your nose and into your mouth.

Do not eat, drink, smoke, clean your teeth, chew gum, or use nasal sprays for 30 minutes before collecting your saliva sample.

TEMPUS + SALIVADIRECT™

FUNNEL COLLECTION KIT

TEMPUS

600 West Chicago Ave, Ste 510
Chicago, IL 60654
Tempus.com | 800.739.4137

Tempus Labs, Inc. | All Content Copyright 2020 | 12.22.20

PRE-COLLECTION

This test is designed to test saliva that naturally collects in your mouth (for example when you think about a delicious meal).

WARNING: Do not eat, drink, smoke, clean your teeth, chew gum, or use nasal sprays for 30 minutes before collecting your saliva sample.

1. Visit the FedEx website at **fedex.com/labreturns** to view drop box locations and pick up schedules.
   - Your sample should be shipped the same day that you collect it. Make sure you can get to your drop box before the last pickup time on the same day you collect your sample.
   - Do not deliver your sample to a drop box on the weekend.
2. Identify your sample tube.
   - Using a permanent pen, write your date of birth (MM-DD-YY) on the patient identifier sticker.
   - Remove the adhesive backing from the patient identifier sticker and affix it to the sample transport tube.
   - Do not write on or cover up the printed barcode or ID number on the tube.
3. Activate your kit at **tinyurl.com/SalivaDirect2**.
   - Follow the instructions on the page to fill in your information.

COLLECTING YOUR SAMPLE

4. Wash your hands with soap and water for at least 20 seconds or clean them with alcohol-based sanitizer.
5. Remove everything from the collection kit and place it in front of you. Set the bag aside. **Avoid touching the inside of the funnel.**
6. Pick up the funnel and the sample tube. Position yourself as shown.

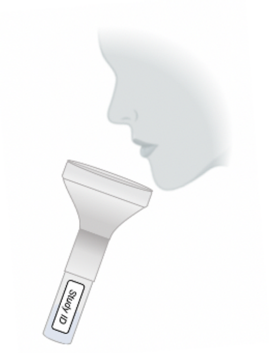

7. Swallow once to clear your mouth.
8. Imagine eating your favorite food, think about an upcoming meal or even a sour lemon and allow saliva to collect in your mouth. Be patient and it will start to collect!
   - Remember to avoid coughing or sniffing.
9. Hold the funnel up so that it surrounds your mouth. Then gently release the saliva that has collected in your mouth into the funnel.
   - Please avoid 'spitting' into the tube. Saliva should instead be gently released from your mouth.
10. Collect enough saliva so the tube is half-filled. Any bubbles in the tube do not count towards the final volume.
    - You may have to repeat this process a couple of times until the tube is half-full.
    - Be patient and don't force it. Just keep thinking about food and you'll get there!
11. Once you have collected enough saliva, remove the funnel from the sample tube and tightly screw the cap back onto the tube.
    - Make sure the cap is secure before proceeding to the next step.
12. Use the alcohol wipe to wipe your hands and the sample tube. DO NOT wipe the printed part of the label.
13. Place the closed sample tube into the biospecimen bag.
14. Discard the alcohol wipe and used funnel.
15. Wash your hands thoroughly with soap and water or clean them with alcohol-based sanitizer.

RETURNING YOUR SAMPLE

16. Drop off at any FedEx drop box location.
    - Please drop off your sample on the same day you collect it and before the last pickup time.
    - Do not deliver your sample to a drop box on the weekend.

The Tempus + SalivaDirect **bulb pipette collection kit.**

WARNING AND PRECAUTIONS BEFORE BEGINNING

Before providing your sample, first carefully read through the instructions below about how to provide a good saliva sample. **Not following the instructions could lead to an incorrect result.**

It is very important that you do not force yourself to produce a saliva sample. Please do not cough anything up from your chest. Please do not sniff anything back from your nose into your mouth.

Do not eat, drink, smoke, clean your teeth, chew gum, or use nasal sprays for 30 minutes before collecting your saliva sample.

TEMPUS + SALIVADIRECT™

BULB PIPETTE COLLECTION KIT

TEMPUS

600 West Chicago Ave, Ste 510
Chicago, IL 60654
Tempus.com | 800.739.4137

Tempus Labs, Inc. | All Content Copyright 2020 | 12.10.20

PRE-COLLECTION

This test is designed to test saliva that naturally collects in your mouth (for example when you think about a delicious meal).

WARNING: Do not eat, drink, smoke, clean your teeth, chew gum, or use nasal sprays for 30 minutes before collecting your saliva sample.

1. Visit the FedEx website at **fedex.com/labreturns** to view drop box locations and pick up schedules.
   - Your sample should be shipped the same day that you collect it. Make sure you can get to your drop box before the last pickup time on the same day you collect your sample.
   - Do not deliver your sample to a drop box on the weekend.
2. Identify your sample tube.
   - Using a permanent pen, write your date of birth (MM-DD-YY) on the patient identifier sticker.
   - Remove the adhesive backing from the patient identifier sticker and affix it to the sample tube.
   - Do not write on or cover up the printed barcode or ID number on the tube.
3. Activate your kit at **tinyurl.com/SalivaDirect2**.

   Follow the instructions on the page to fill in your information.
4. Wash your hands with soap and water for at least 20 seconds or clean them with alcohol-based sanitizer.
5. Remove everything from the collection kit, place in front of you, and set the bag aside.

COLLECTING YOUR SAMPLE

6. Unscrew the cap from the tube.
7. Place the open tube in front of you so that it is standing upright. You may place the cap to the side for now.
8. Open the bulb pipette packaging **starting at the end with the bulb** (see illustration).

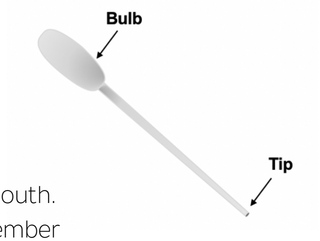

9. Swallow once to clear your mouth.
10. Imagine eating your favorite food, think about an upcoming meal or even a sour lemon and allow saliva to collect in your mouth. Be patient and it will start to collect! Remember to avoid coughing or sniffing.
11. Pick up the bulb pipette and press the bulb end between your index finger and thumb to push out all of the air.
12. Keeping your lips mostly closed, gently insert the tip into your mouth where saliva is collecting.
13. Gently release your finger and thumb to allow the bulb pipette to fill with saliva.
    - Please avoid 'spitting'. Saliva should be gently released from the mouth.
14. Remove the bulb pipette from your mouth and pick up the sample tube with your other hand.
15. Carefully insert the tip of the bulb pipette into the sample tube. Then gently push down on the bulb to slowly empty the saliva into the tube.
16. Repeat steps 10-15 until the sample tube is about half filled. Any bubbles in the tube do not count towards the final volume.
    - You may have to repeat this process a couple of times until the tube is half-full.
    - Be patient and don't force it. Just keep thinking about food and you'll get there!
17. Once you have collected enough saliva, tightly screw the cap back onto the sample tube.
    - Make sure the cap is secure before proceeding to the next step.
18. Use the alcohol wipe to wipe your hands and the sample tube. DO NOT wipe the printed part of the label.
19. Place the closed sample tube into the biospecimen bag.
20. Discard the alcohol wipe and used bulb pipette.
21. Wash your hands thoroughly with soap and water or clean them with alcohol-based sanitizer.

RETURNING YOUR SAMPLE

22. Drop off at any FedEx drop box location.
    - Please drop off your sample on the same day you collect it and before the last pickup time.
    - Do not deliver your sample to a drop box on the weekend.

Welcome to the Tempus COVID-19 collection kit.

Please read through this entire document. If you have any questions, please refer to **tempus.com/faq**.

1. ACTIVATE YOUR KIT

Before collecting your sample, activate your kit online at **tinyurl.com/SalivaDirect2**. Follow the instructions on the page to fill in the necessary contact information to tie your sample to your results.

2. COLLECT YOUR SAMPLE

Collection your sample following the full instructions. **Do not eat, drink, smoke, clean your teeth, chew gum, or use nasal sprays for 30 minutes before collecting your saliva sample.**

3. SHIP BACK YOUR SAMPLE SAME DAY

Your sample should be shipped the same day it is collected. See the collection brochure for more information.

TEMPUS

COMPONENTS

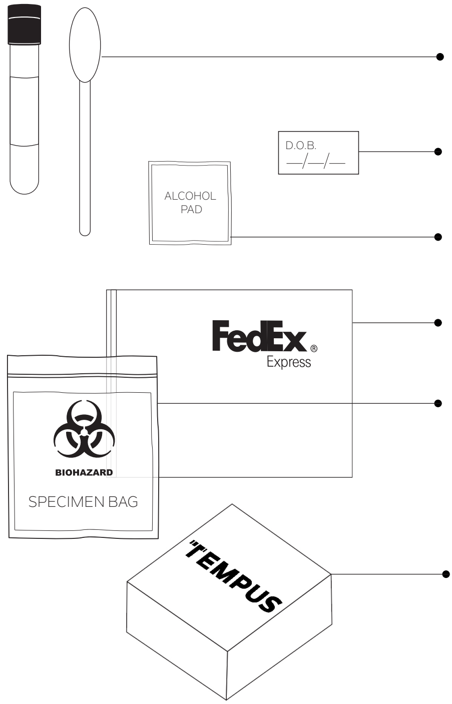

Labeled screw-cap tube with bulb pipette

Patient identifier sticker

Alcohol pad

Pre-labeled FedEx Clinical Pak

Biohazard collection bag with ziploc seal

(absorbent sheet should stay in bag)

Tempus box for return shipment

Note: Make sure to keep this box to use when returning your sample.

REMINDERS & TIPS

Make sure you are able to your drop box before the last pickup time on the same day you collect your sample. Do not deliver your sample to a drop box on the weekend.

Use a permanent pen or marker when labeling your collection tube.

This test has not been FDA cleared or approved, but has been authorized for emergency use by FDA under an EUA for use by authorized laboratories; This test has been authorized only for the detection of nucleic acid from SARSCoV-2, influenza A virus, and influenza B virus, and not for any other viruses or pathogens; and The emergency use of this test is only authorized for the duration of the declaration that circumstances exist justifying the authorization of emergency use of in vitro diagnostics for detection and/or diagnosis of COVID-19 under Section 564(b)(1) of the Federal Food, Drug, and Cosmetic Act, 21 U.S.C. § 360bbb-3(b)(1)

Welcome to the Tempus COVID-19 collection kit.

Please read through this entire document. If you have any questions, please refer to **tempus.com/faq**.

1. ACTIVATE YOUR KIT

Before collecting your sample, activate your kit online at **tinyurl.com/SalivaDirect2**. Follow the instructions on the page to fill in the necessary contact information to tie your sample to your results.

2. COLLECT YOUR SAMPLE

Collection your sample following the full instructions. **Do not eat, drink, smoke, clean your teeth, chew gum, or use nasal sprays for 30 minutes before collecting your saliva sample.**

3. SHIP BACK YOUR SAMPLE SAME DAY

Your sample should be shipped the same day it is collected. See the collection brochure for more information.

TEMPUS

COMPONENTS

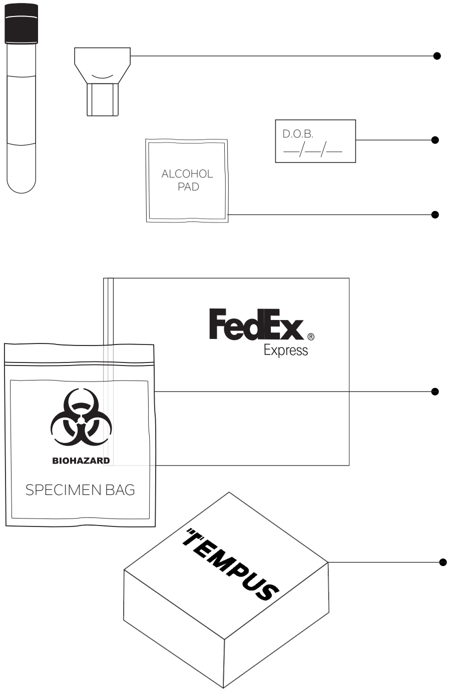

Labeled screw-cap tube with funnel

Patient identifier sticker

Alcohol pad

Pre-labeled FedEx Clinical Pak

Biohazard collection bag with ziploc seal

(absorbent sheet should stay in bag)

Tempus box for return shipment

Note: Make sure to keep this box to use when returning your sample.

REMINDERS & TIPS

Make sure you are able to your drop box before the last pickup time on the same day you collect your sample. Do not deliver your sample to a drop box on the weekend.

Use a permanent pen or marker when labeling your collection tube.

This test has not been FDA cleared or approved, but has been authorized for emergency use by FDA under an EUA for use by authorized laboratories; This test has been authorized only for the detection of nucleic acid from SARSCoV-2, influenza A virus, and influenza B virus, and not for any other viruses or pathogens; and The emergency use of this test is only authorized for the duration of the declaration that circumstances exist justifying the authorization of emergency use of in vitro diagnostics for detection and/or diagnosis of COVID-19 under Section 564(b)(1) of the Federal Food, Drug, and Cosmetic Act, 21 U.S.C. § 360bbb-3(b)(1)
